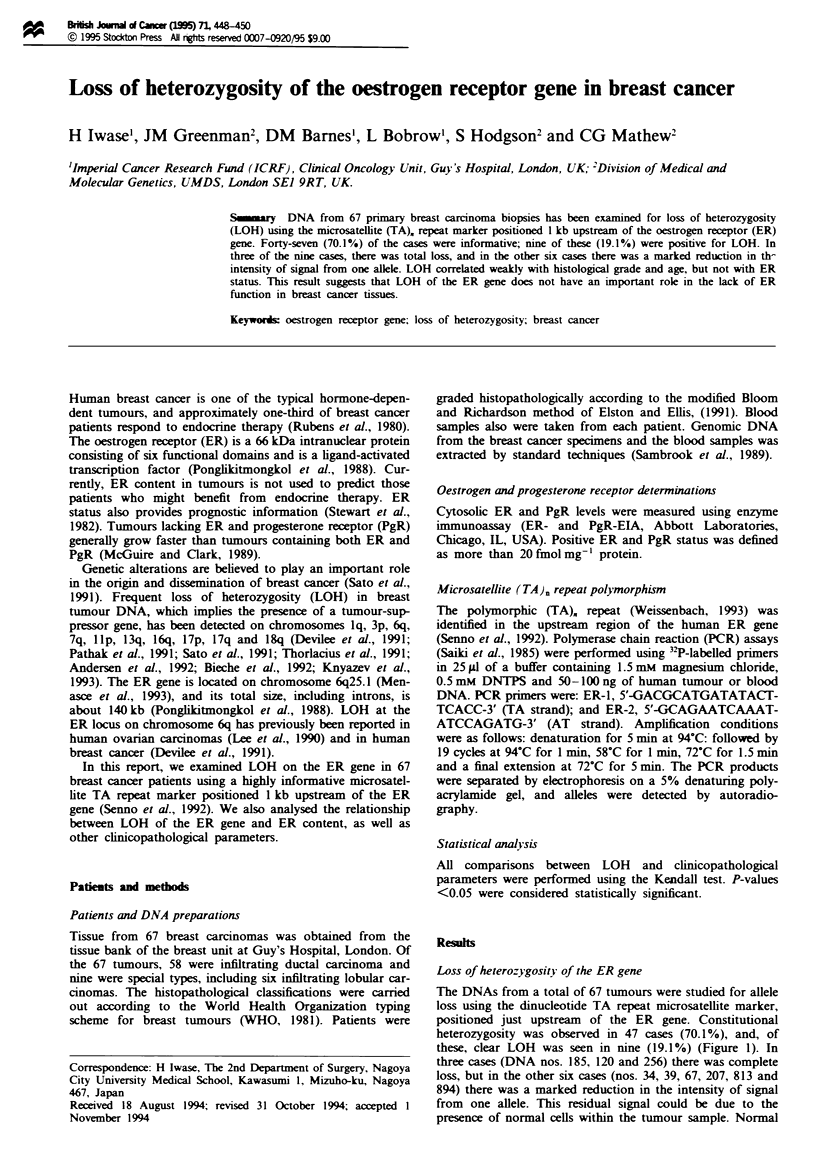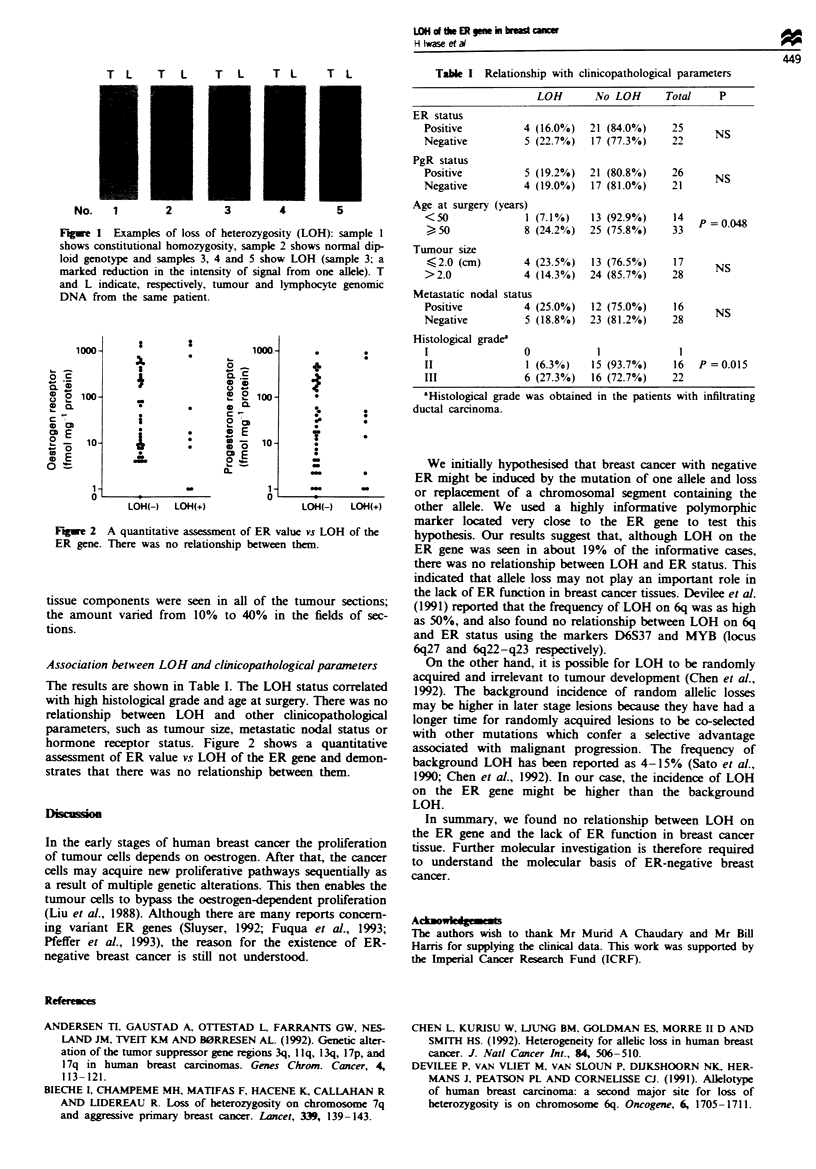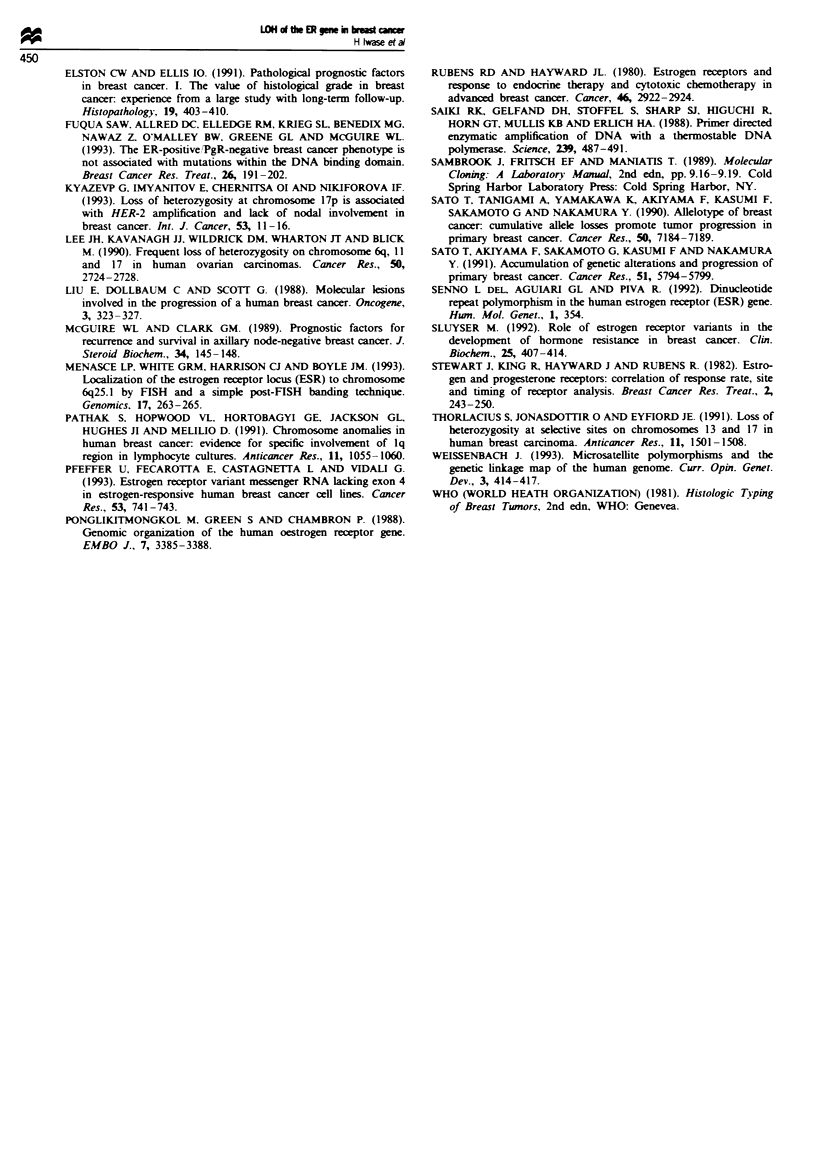# Loss of heterozygosity of the oestrogen receptor gene in breast cancer.

**DOI:** 10.1038/bjc.1995.91

**Published:** 1995-03

**Authors:** H. Iwase, J. M. Greenman, D. M. Barnes, L. Bobrow, S. Hodgson, C. G. Mathew

**Affiliations:** Imperial Cancer Research Fund (ICRF), Guy's Hospital, London, UK.

## Abstract

**Images:**


British Journal of Cancer (1995) 71, 448–450

© 1995 Stockton Press All rights reserved 0007–0920/95 $9.00

### Loss of heterozygosity of the oestrogen receptor gene in breast cancer

H Iwase[1], JM Greenman[2], DM Barnes[1], L Bobrow[1], S Hodgson[2] and CG Mathew[2]

[1]*Imperial Cancer Research Fund (ICRF), Clinical Oncology Unit, Guy's Hospital, London, UK;* [2]*Division of Medical and Molecular Genetics, UMDS, London SE1 9RT, UK.*

**Summary** DNA from 67 primary breast carcinoma biopsies has been examined for loss of heterozygosity (LOH) using the microsatellite $(TA)_n$ repeat marker positioned 1 kb upstream of the oestrogen receptor (ER) gene. Forty-seven (70.1%) of the cases were informative; nine of these (19.1%) were positive for LOH. In three of the nine cases, there was total loss, and in the other six cases there was a marked reduction in th^ intensity of signal from one allele. LOH correlated weakly with histological grade and age, but not with ER status. This result suggests that LOH of the ER gene does not have an important role in the lack of ER function in breast cancer tissues.

**Keywords:** oestrogen receptor gene; loss of heterozygosity; breast cancer

Human breast cancer is one of the typical hormone-dependent tumours, and approximately one-third of breast cancer patients respond to endocrine therapy (Rubens *et al.*, 1980). The oestrogen receptor (ER) is a 66 kDa intranuclear protein consisting of six functional domains and is a ligand-activated transcription factor (Ponglikitmongkol *et al.*, 1988). Currently, ER content in tumours is not used to predict those patients who might benefit from endocrine therapy. ER status also provides prognostic information (Stewart *et al.*, 1982). Tumours lacking ER and progesterone receptor (PgR) generally grow faster than tumours containing both ER and PgR (McGuire and Clark, 1989).

Genetic alterations are believed to play an important role in the origin and dissemination of breast cancer (Sato *et al.*, 1991). Frequent loss of heterozygosity (LOH) in breast tumour DNA, which implies the presence of a tumour-suppressor gene, has been detected on chromosomes 1q, 3p, 6q, 7q, 11p, 13q, 16q, 17p, 17q and 18q (Devilee *et al.*, 1991; Pathak *et al.*, 1991; Sato *et al.*, 1991; Thorlacius *et al.*, 1991; Andersen *et al.*, 1992; Bieche *et al.*, 1992; Knyazev *et al.*, 1993). The ER gene is located on chromosome 6q25.1 (Menasce *et al.*, 1993), and its total size, including introns, is about 140 kb (Ponglikitmongkol *et al.*, 1988). LOH at the ER locus on chromosome 6q has previously been reported in human ovarian carcinomas (Lee *et al.*, 1990) and in human breast cancer (Devilee *et al.*, 1991).

In this report, we examined LOH on the ER gene in 67 breast cancer patients using a highly informative microsatellite TA repeat marker positioned 1 kb upstream of the ER gene (Senno *et al.*, 1992). We also analysed the relationship between LOH of the ER gene and ER content, as well as other clinicopathological parameters.

### Patients and methods

### *Patients and DNA preparations*

Tissue from 67 breast carcinomas was obtained from the tissue bank of the breast unit at Guy's Hospital, London. Of the 67 tumours, 58 were infiltrating ductal carcinoma and nine were special types, including six infiltrating lobular carcinomas. The histopathological classifications were carried out according to the World Health Organization typing scheme for breast tumours (WHO, 1981). Patients were graded histopathologically according to the modified Bloom and Richardson method of Elston and Ellis, (1991). Blood samples also were taken from each patient. Genomic DNA from the breast cancer specimens and the blood samples was extracted by standard techniques (Sambrook *et al.*, 1989).

### *Oestrogen and progesterone receptor determinations*

Cytosolic ER and PgR levels were measured using enzyme immunoassay (ER- and PgR-EIA, Abbott Laboratories, Chicago, IL, USA). Positive ER and PgR status was defined as more than 20 fmol $mg^{-1}$ protein.

### *Microsatellite $(TA)_n$ repeat polymorphism*

The polymorphic $(TA)_n$ repeat (Weissenbach, 1993) was identified in the upstream region of the human ER gene (Senno *et al.*, 1992). Polymerase chain reaction (PCR) assays (Saiki *et al.*, 1985) were performed using $^{32}P$-labelled primers in 25 µl of a buffer containing 1.5 mM magnesium chloride, 0.5 mM DNTPS and 50–100 ng of human tumour or blood DNA. PCR primers were: ER-1, 5′-GACGCATGATATACT-TCACC-3′ (TA strand); and ER-2, 5′-GCAGAATCAAAT-ATCCAGATG-3′ (AT strand). Amplification conditions were as follows: denaturation for 5 min at 94°C: followed by 19 cycles at 94°C for 1 min, 58°C for 1 min, 72°C for 1.5 min and a final extension at 72°C for 5 min. The PCR products were separated by electrophoresis on a 5% denaturing polyacrylamide gel, and alleles were detected by autoradiography.

### *Statistical analysis*

All comparisons between LOH and clinicopathological parameters were performed using the Kendall test. *P*-values $<0.05$ were considered statistically significant.

### Results

### *Loss of heterozygosity of the ER gene*

The DNAs from a total of 67 tumours were studied for allele loss using the dinucleotide TA repeat microsatellite marker, positioned just upstream of the ER gene. Constitutional heterozygosity was observed in 47 cases (70.1%), and, of these, clear LOH was seen in nine (19.1%) (Figure 1). In three cases (DNA nos. 185, 120 and 256) there was complete loss, but in the other six cases (nos. 34, 39, 67, 207, 813 and 894) there was a marked reduction in the intensity of signal from one allele. This residual signal could be due to the presence of normal cells within the tumour sample. Normal

Correspondence: H Iwase, The 2nd Department of Surgery, Nagoya City University Medical School, Kawasumi 1, Mizuho-ku, Nagoya 467, Japan
Received 18 August 1994; revised 31 October 1994; accepted 1 November 1994

**Figure 1** Examples of loss of heterozygosity (LOH): sample 1 shows constitutional homozygosity, sample 2 shows normal diploid genotype and samples 3, 4 and 5 show LOH (sample 3; a marked reduction in the intensity of signal from one allele). T and L indicate, respectively, tumour and lymphocyte genomic DNA from the same patient.

**Figure 2** A quantitative assessment of ER value *vs* LOH of the ER gene. There was no relationship between them.

tissue components were seen in all of the tumour sections; the amount varied from 10% to 40% in the fields of sections.

### *Association between LOH and clinicopathological parameters*

The results are shown in Table I. The LOH status correlated with high histological grade and age at surgery. There was no relationship between LOH and other clinicopathological parameters, such as tumour size, metastatic nodal status or hormone receptor status. Figure 2 shows a quantitative assessment of ER value *vs* LOH of the ER gene and demonstrates that there was no relationship between them.

**Table I** Relationship with clinicopathological parameters

| | *LOH* | *No LOH* | *Total* | P |
|---|---|---|---|---|
| ER status | | | | |
| Positive | 4 (16.0%) | 21 (84.0%) | 25 | NS |
| Negative | 5 (22.7%) | 17 (77.3%) | 22 | |
| PgR status | | | | |
| Positive | 5 (19.2%) | 21 (80.8%) | 26 | NS |
| Negative | 4 (19.0%) | 17 (81.0%) | 21 | |
| Age at surgery (years) | | | | |
| <50 | 1 (7.1%) | 13 (92.9%) | 14 | $P = 0.048$ |
| ⩾50 | 8 (24.2%) | 25 (75.8%) | 33 | |
| Tumour size | | | | |
| ⩽2.0 (cm) | 4 (23.5%) | 13 (76.5%) | 17 | NS |
| >2.0 | 4 (14.3%) | 24 (85.7%) | 28 | |
| Metastatic nodal status | | | | |
| Positive | 4 (25.0%) | 12 (75.0%) | 16 | NS |
| Negative | 5 (18.8%) | 23 (81.2%) | 28 | |
| Histological grade[a] | | | | |
| I | 0 | 1 | 1 | |
| II | 1 (6.3%) | 15 (93.7%) | 16 | $P = 0.015$ |
| III | 6 (27.3%) | 16 (72.7%) | 22 | |

[a]Histological grade was obtained in the patients with infiltrating ductal carcinoma.

### Discussion

In the early stages of human breast cancer the proliferation of tumour cells depends on oestrogen. After that, the cancer cells may acquire new proliferative pathways sequentially as a result of multiple genetic alterations. This then enables the tumour cells to bypass the oestrogen-dependent proliferation (Liu *et al.*, 1988). Although there are many reports concerning variant ER genes (Sluyser, 1992; Fuqua *et al.*, 1993; Pfeffer *et al.*, 1993), the reason for the existence of ER-negative breast cancer is still not understood.

We initially hypothesised that breast cancer with negative ER might be induced by the mutation of one allele and loss or replacement of a chromosomal segment containing the other allele. We used a highly informative polymorphic marker located very close to the ER gene to test this hypothesis. Our results suggest that, although LOH on the ER gene was seen in about 19% of the informative cases, there was no relationship between LOH and ER status. This indicated that allele loss may not play an important role in the lack of ER function in breast cancer tissues. Devilee *et al.* (1991) reported that the frequency of LOH on 6q was as high as 50%, and also found no relationship between LOH on 6q and ER status using the markers D6S37 and MYB (locus 6q27 and 6q22-q23 respectively).

On the other hand, it is possible for LOH to be randomly acquired and irrelevant to tumour development (Chen *et al.*, 1992). The background incidence of random allelic losses may be higher in later stage lesions because they have had a longer time for randomly acquired lesions to be co-selected with other mutations which confer a selective advantage associated with malignant progression. The frequency of background LOH has been reported as 4–15% (Sato *et al.*, 1990; Chen *et al.*, 1992). In our case, the incidence of LOH on the ER gene might be higher than the background LOH.

In summary, we found no relationship between LOH on the ER gene and the lack of ER function in breast cancer tissue. Further molecular investigation is therefore required to understand the molecular basis of ER-negative breast cancer.

**Acknowledgements**
The authors wish to thank Mr Murid A Chaudary and Mr Bill Harris for supplying the clinical data. This work was supported by the Imperial Cancer Research Fund (ICRF).

### References

ANDERSEN TI, GAUSTAD A, OTTESTAD L, FARRANTS GW, NESLAND JM, TVEIT KM AND BØRRESEN AL. (1992). Genetic alteration of the tumor suppressor gene regions 3q, 11q, 13q, 17p, and 17q in human breast carcinomas. *Genes Chrom. Cancer*, **4**, 113–121.

BIECHE I, CHAMPEME MH, MATIFAS F, HACENE K, CALLAHAN R AND LIDEREAU R. Loss of heterozygosity on chromosome 7q and aggressive primary breast cancer. *Lancet*, **339**, 139–143.

CHEN L, KURISU W, LJUNG BM, GOLDMAN ES, MORRE II D AND SMITH HS. (1992). Heterogeneity for allelic loss in human breast cancer. *J. Natl Cancer Int.*, **84**, 506–510.

DEVILEE P, VAN VLIET M, VAN SLOUN P, DIJKSHOORN NK, HERMANS J, PEATSON PL AND CORNELISSE CJ. (1991). Allelotype of human breast carcinoma: a second major site for loss of heterozygosity is on chromosome 6q. *Oncogene*, **6**, 1705–1711.

ELSTON CW AND ELLIS IO. (1991). Pathological prognostic factors in breast cancer. I. The value of histological grade in breast cancer: experience from a large study with long-term follow-up. *Histopathology*, **19**, 403-410.

FUQUA SAW, ALLRED DC, ELLEDGE RM, KRIEG SL, BENEDIX MG, NAWAZ Z, O'MALLEY BW, GREENE GL AND McGUIRE WL. (1993). The ER-positive/PgR-negative breast cancer phenotype is not associated with mutations within the DNA binding domain. *Breast Cancer Res. Treat.*, **26**, 191-202.

KYAZEVP G, IMYANITOV E, CHERNITSA OI AND NIKIFOROVA IF. (1993). Loss of heterozygosity at chromosome 17p is associated with *HER*-2 amplification and lack of nodal involvement in breast cancer. *Int. J. Cancer*, **53**, 11-16.

LEE JH, KAVANAGH JJ, WILDRICK DM, WHARTON JT AND BLICK M. (1990). Frequent loss of heterozygosity on chromosome 6q, 11 and 17 in human ovarian carcinomas. *Cancer Res.*, **50**, 2724-2728.

LIU E, DOLLBAUM C AND SCOTT G. (1988). Molecular lesions involved in the progression of a human breast cancer. *Oncogene*, **3**, 323-327.

McGUIRE WL AND CLARK GM. (1989). Prognostic factors for recurrence and survival in axillary node-negative breast cancer. *J. Steroid Biochem.*, **34**, 145-148.

MENASCE LP, WHITE GRM, HARRISON CJ AND BOYLE JM. (1993). Localization of the estrogen receptor locus (ESR) to chromosome 6q25.1 by FISH and a simple post-FISH banding technique. *Genomics*, **17**, 263-265.

PATHAK S, HOPWOOD VL, HORTOBAGYI GE, JACKSON GL, HUGHES JI AND MELILIO D. (1991). Chromosome anomalies in human breast cancer: evidence for specific involvement of 1q region in lymphocyte cultures. *Anticancer Res.*, **11**, 1055-1060.

PFEFFER U, FECAROTTA E, CASTAGNETTA L AND VIDALI G. (1993). Estrogen receptor variant messenger RNA lacking exon 4 in estrogen-responsive human breast cancer cell lines. *Cancer Res.*, **53**, 741-743.

PONGLIKITMONGKOL M, GREEN S AND CHAMBRON P. (1988). Genomic organization of the human oestrogen receptor gene. *EMBO J.*, **7**, 3385-3388.

RUBENS RD AND HAYWARD JL. (1980). Estrogen receptors and response to endocrine therapy and cytotoxic chemotherapy in advanced breast cancer. *Cancer*, **46**, 2922-2924.

SAIKI RK, GELFAND DH, STOFFEL S, SHARP SJ, HIGUCHI R, HORN GT, MULLIS KB AND ERLICH HA. (1988). Primer directed enzymatic amplification of DNA with a thermostable DNA polymerase. *Science*, **239**, 487-491.

SAMBROOK J, FRITSCH EF AND MANIATIS T. (1989). *Molecular Cloning: A Laboratory Manual*, 2nd edn, pp. 9.16-9.19. Cold Spring Harbor Laboratory Press: Cold Spring Harbor, NY.

SATO T, TANIGAMI A, YAMAKAWA K, AKIYAMA F, KASUMI F, SAKAMOTO G AND NAKAMURA Y. (1990). Allelotype of breast cancer: cumulative allele losses promote tumor progression in primary breast cancer. *Cancer Res.*, **50**, 7184-7189.

SATO T, AKIYAMA F, SAKAMOTO G, KASUMI F AND NAKAMURA Y. (1991). Accumulation of genetic alterations and progression of primary breast cancer. *Cancer Res.*, **51**, 5794-5799.

SENNO L DEL, AGUIARI GL AND PIVA R. (1992). Dinucleotide repeat polymorphism in the human estrogen receptor (ESR) gene. *Hum. Mol. Genet.*, **1**, 354.

SLUYSER M. (1992). Role of estrogen receptor variants in the development of hormone resistance in breast cancer. *Clin. Biochem.*, **25**, 407-414.

STEWART J, KING R, HAYWARD J AND RUBENS R. (1982). Estrogen and progesterone receptors: correlation of response rate, site and timing of receptor analysis. *Breast Cancer Res. Treat.*, **2**, 243-250.

THORLACIUS S, JONASDOTTIR O AND EYFIORD JE. (1991). Loss of heterozygosity at selective sites on chromosomes 13 and 17 in human breast carcinoma. *Anticancer Res.*, **11**, 1501-1508.

WEISSENBACH J. (1993). Microsatellite polymorphisms and the genetic linkage map of the human genome. *Curr. Opin. Genet. Dev.*, **3**, 414-417.

WHO (WORLD HEATH ORGANIZATION) (1981). *Histologic Typing of Breast Tumors*, 2nd edn, WHO: Genevea.